# Rheological Properties, Surface Microhardness, and Dentin Shear Bond Strength of Resin-Modified Glass Ionomer Cements Containing Methacrylate-Functionalized Polyacids and Spherical Pre-Reacted Glass Fillers

**DOI:** 10.3390/jfb12030042

**Published:** 2021-07-14

**Authors:** Whithipa Thepveera, Wisitsin Potiprapanpong, Arnit Toneluck, Somruethai Channasanon, Chutikarn Khamsuk, Naruporn Monmaturapoj, Siriporn Tanodekaew, Piyaphong Panpisut

**Affiliations:** 1 Faculty of Dentistry, Thammasat University, Pathum Thani 12120, Thailand; whithipa.the@dome.tu.ac.th (W.T.); wisitsin.pot@dome.tu.ac.th (W.P.); rnit_pook@hotmail.com (A.T.); 2 National Metal and Materials Technology Center (MTEC), National Science and Technology Development Agency, Pathum Thani 12120, Thailand; somruec@mtec.or.th (S.C.); siriporn@mtec.or.th (S.T.); 3 Assistive Technology and Medical Devices Research Center (A-MED), National Science and Technology Development Agency, Pathum Thani 12120, Thailand; chutikarn.kha@ncr.nstda.or.th (C.K.); naruporn.mon@nstda.or.th (N.M.); 4 Thammasat University Research Unit in Dental and Bone Substitute Biomaterials, Thammasat University, Pathum Thani 12120, Thailand

**Keywords:** resin-modified glass ionomer cements, shear bond strength, 2-hydroxyethyl methacrylate, surface microhardness, rheological properties

## Abstract

The aim of this study was to prepare experimental resin-modified glass ionomer cements (RMGICs) containing low levels of hydroxyethyl methacrylate (HEMA) for pulp protection. Liquid and powder phases of the experimental RMGICs were polyacid functionalized with methacrylate groups and spherical pre-reacted glass fillers (SPG). Two types of liquid phase containing 0 wt. % HEMA (CM liquid) or 5 wt. % HEMA (CMH liquid) were formulated. The experimental RMGICs were prepared by mixing SPG fillers with CM liquid (F1) or CMH liquid (F2). Rheological properties were examined using a strain-controlled rheometer (n = 5). The Vickers microhardness (n = 5) and dentin shear bond strength (SBS) (n = 10) of the materials were tested. Commercial pulp protection materials (Vitrebond and TheraCal LC) were used as comparisons. The viscosity and surface microhardness of F1 (22 m Pa·s, 18 VHN) and F2 (18 m Pa·s, 16 VHN) were significantly higher than those of Vitrebond (6 mPa·s, 6 VHN) and TheraCal (0.1 mPa·s, 7 VHN). The SBS of F1 (10.7 MPa) and F2 (11.9 MPa) was comparable to that of Vitrebond (15.4 MPa) but higher than that of TheraCal LC (5.6 MPa). The addition of 5 wt. % HEMA showed no significant effect on viscosity, surface microhardness, or SBS of the experimental RMGICs. The experimental materials showed higher viscosity and microhardness but similar SBS when compared with the commercial RMGIC.

## 1. Introduction

Untreated dental caries remains the most common preventable oral disease affecting people globally at all ages [1]. Carious lesions may progress and become uncleanable cavities that require restorative treatments. The current minimally invasive technique for managing deep carious lesions is the selective caries removal technique. The technique involves total caries removal at peripheral areas whilst leaving the demineralized dentin (firm/soft dentin) at the deep pulpal area to reduce the risk of accidental pulpal exposure [2,3]. The application of cavity liners over the remaining demineralized dentin near the pulp has been considered an essential step for restorative treatment [4]. A clinical trial indicated that the success of the selective caries removal technique at 12 months was not associated with the placement of liner over the demineralized dentin [5]. However, a recent survey demonstrated that 58% of dental practitioners who performed the selective caries removal technique preferred to place pulp protection materials or liner over the demineralized dentin prior to the placement of definitive restorations [6]. 

Pulp protection materials or liners should exhibit a good flow to enhance the intimate adaptation between materials and the cavity floor [7]. This could potentially help to reduce gap formation at the tooth–restoration interface. Additionally, the high surface hardness of the materials is also required to ensure that the materials can withstand mechanical forces or acid etching during the placement of the definitive coronal restorations [8]. Furthermore, the strong chemical/mechanical adhesions with dentin are essential to help reduce the risk of debonding at the tooth–material interface due to polymerization shrinkage [9].

Resin-modified glass ionomer cement (RMGIC) is one of the most commonly used pulp protection materials [10]. The attractive properties of RMGICs include the command setting, fluoride ion release, and the chemical/mechanical bond to dentin. The materials set via an acid-base reaction and light-activated free radical polymerization. The main composition of the powder phase is fluoroaluminosilicate glass. The liquid phase contains a copolymer of polyacrylic acids; a resin monomer, such as 2-hydroxyethyl methacrylate (HEMA); a photoinitiator [11]. RMGICs can bond to dentin by two main mechanisms. The first mechanism is chemical adhesion between anion of polyacrylic acids and calcium ions of mineral apatite in dentin. The second mechanism is micromechanical retention. The self-etching characteristic of RMGICs enable the formation of a shallow hybrid layer with the conditioned dentin [12]. 

The major concern of RMGICs is the risk of releasing unreacted and low molecular weight HEMA (130.14 g/mol). Various in vitro studies revealed the toxic effects of HEMA on human cells [13,14,15]. Additionally, poor waste management in dental practices may cause the monomer leach out, which could contaminate the environment [16]. The current commercial RMGIC for pulp protection contains high level of HEMA (20–30 wt. %). Therefore, several studies developed RMGICs that can be cured by free-radical polymerization without the addition of methacrylate monomers to reduce the need for HEMA. This can be achieved by the incorporation of methacrylate moieties onto the polyacrylic backbone [17,18,19]. However, HEMA contains the hydrophilic (OH^−^) group, which could act as an adhesion-promoting agent to moist dentin [20]. In addition, the lack of HEMA may negatively affect the viscosity, flowability, and setting, which could compromise the handling characteristics of the materials. Previous studies introduced the use of spherical glass pre-reacted glass fillers (SPG) as the powder phase for experimental conventional glass ionomer cements [21,22]. The experimental materials exhibited comparable strength to that of commercial materials. The use of SPG fillers may promote polymer cross-linking, which may help increase the mechanical properties of the glass ionomer cements [23].

The objective of this study was therefore to prepare RMGICs using polyacid functionalized with methacrylate groups and spherical pre-reacted glass fillers as the liquid phase and powder phase, respectively. The effects of using two different liquid formulations (0 wt. % or 5 wt. % HEMA) on rheological properties, surface microhardness, and dentin shear bond strength of the materials were examined. The first null hypothesis of the current study was that the use of different liquid phase does not affect the tested properties of the experimental materials. The second hypothesis was that the rheological properties, surface microhardness, and dentin shear bond strength of the experimental materials do not significantly differ from those of the commercial counterparts. 

## 2. Material and Methods

### 2.1. Material Preparation 

Polyacids were prepared using the following chemicals: acrylic acid (Acros Organics, Fair Lawn, NJ, USA); maleic acid (Sigma-Aldrich, St. Louis, MO, USA); tartaric acid (Sigma-Aldrich); glycidyl methacrylate (Sigma-Aldrich); potassium persulphate (Fluka Analytical, Honeywell, Charlotte, NC, USA); butylated hydroxytoluene (Fluka Analytical), isopropanol (RCI Labscan Limited, Bangkok, Thailand); pyridine (RCI Labscan Limited); diethyl ether (RCI Labscan Limited); tetrahydrofuran (RCI Labscan Limited). 

**Figure 1:**
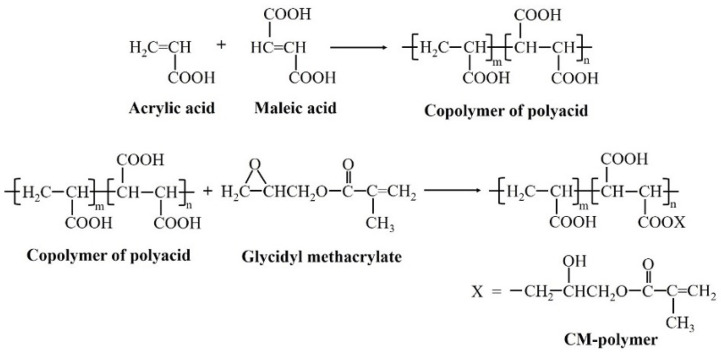
Illustration of the synthesis process of the CM polymer.

The liquid phase for the experimental RMGICs was prepared according to the protocol used in a previously published study [17] (Figure 1). Firstly, the copolymer of acrylic acid and maleic acid with a 4:1 feed molar ratio was synthesized in an aqueous solution using potassium persulphate as an initiator and isopropanol as a chain-transfer agent. The reaction was carried out under nitrogen atmosphere at 80 °C for 4 h. The synthesized copolymer was then concentrated using a rotary evaporator (Büchi Rotavapor R-114, Büchi Lab., Flawil, Switzerland) followed by the drying process using a lyophilizer (Supermodulyo-230, Thermo Fisher Scientific, Waltham, MA, USA). 

For the methacrylation process, the copolymer was reacted with glycidyl methacrylate (GMA) in THF at 60 °C for 5 h under nitrogen gas. Pyridine and butylated hydroxytoluene were used as a catalyst and an inhibitor, respectively. The final product (CM polymer) was precipitated in diethyl ether and dried in a vacuum oven at room temperature. The molar mass of the CM polymer determined by Gel Permeation Chromatography (Water 600E, Waters Corp., Milford, MA, USA) was approximately 55,000 Dalton. Two formulations of liquid phase containing 0 wt. % (CM) or 5 wt. % HEMA (CMH) were prepared (Table 1).

**Figure 2:**
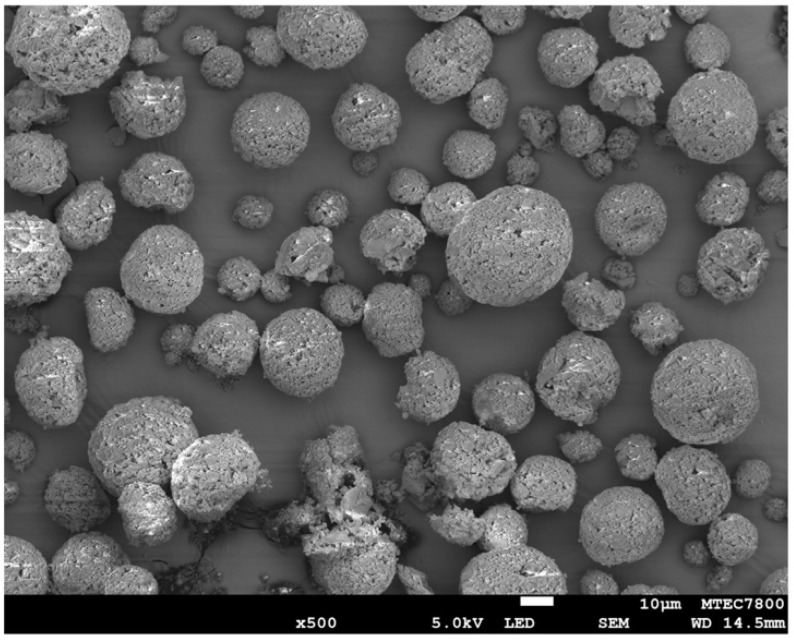
SEM image of spherical pre-reacted glass fillers. Reproduced with permission under Creative Commons Attribution License from Panpisut et al., 2020. Reprinted with permission from ref. [21].

The fluoroaluminosilicate glass (SiO_2_-Al_2_O_3_-CaF_2_-ZrO_2_) was produced according to methods described in previous studies [21,22]. Briefly, the prepared glass was mixed with 2 wt. % CM liquid to produce the pre-reacted glass. The pre-reacted glass was ground for 3 h and subsequently sprayed using a spray dryer to produce spherical glass fillers (particle diameter ~10–20 μm). The obtained glasses with irregular shape and spherical shape were mixed at 60:40 by weight to produced SPG fillers (Figure 2). 

The powder phase and liquid phase of RMGICs were initially analyzed using an FTIR-ATR (Nicolet iS5, Thermo Fisher Scientific, Waltham, MA, USA). FTIR spectra at the region of from 700 to 4000 cm^−1^ with a resolution of 8 cm^−1^ were recorded.

**Figure 3:**
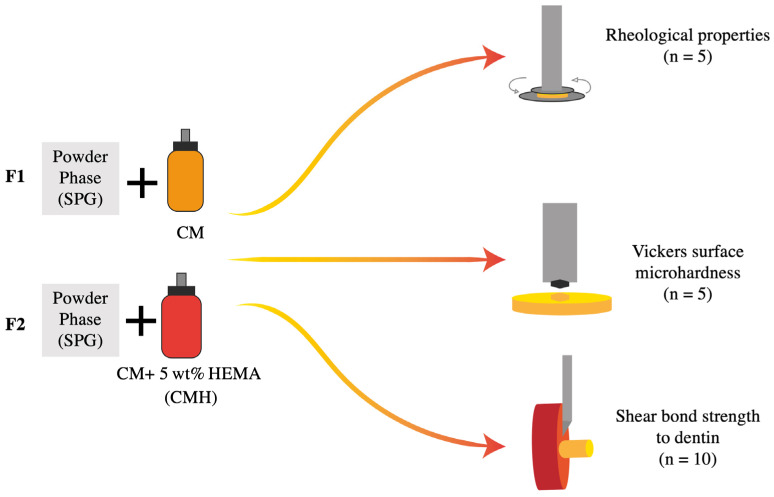
Illustration of the methods used in the current study.

The powder and liquid were weighed using a four-figure balance (MS-DNY-43, METTLER TOLEDO, Columbus, OH, USA) and hand-mixed using a plastic spatula with mixing pad within 20 s. The experimental RMGICs were prepared using CM or CMH liquid. Commercial pulp protection materials, including RMGIC (Vitrebond, VB) and resin-modified Ca-Si cement (TheraCal LC, TC) (Table 2), were used for comparison. The commercial materials were prepared according to the manufacturer’s instructions. The testing methods used in the current study are provided in Figure 3. 

### 2.2. Rheological Test

A strain-controlled rheometer (Thermo Scientific™ HAAKE™ MARS™, Thermo Fisher Scientific Inc., Karlsruhe, Germany) was employed to assess the rheological properties of the materials (n = 5). Powder and liquid phases were weighted using a four-figure balance and hand-mixed using a plastic spatula for 20 s. The specimens were then placed between two parallel circular plates (diameter of 10 mm) with a gap of 0.5 mm. The test was performed at a controlled temperature of 37 °C. The time sweep measurement was tested using the oscillatory frequency and strain fixed at 1 rad/s and 0.02%. The test was conducted for 20 min [22]. 

The viscosity of materials (Pa·s) was assessed immediately after mixing. Additionally, the gelation time (s), which was defined as the time at which the storage modulus (G′) reached a similar value to the loss modulus (G″), was also recorded. The gelation time or gel point represents the time when the material transformed from fluid-like behavior to solid-like or elastic behavior [24,25]. 

### 2.3. Vickers Surface Microhardness Testing

Disc specimens (n = 5) were prepared using a metal circlip (0.5 mm × 6 mm, Springmaster Ltd., Redditch, UK). The materials were prepared and placed in the circlips. Then, they were covered by acetate sheets on top and bottom surfaces and compressed with glass slides to remove excess material. They were light-cured using an LED light-curing unit (light intensity of 1100–1330 mW/cm^2^, Demi Ultra, Kavo Kerr Group, Charlotte, NC, USA) for 40 s on the top and bottom surfaces. The specimens were left at room temperature (25 ± 1 °C) for 24 h to allow the completion of polymerization. They were then immersed in a tube containing 5 mL of deionized water. The tubes were incubated at 37 °C for 24 h. Vickers surface microhardness was tested using a microhardness tester (FM-800, Future-Tech Corp, Kanagawa, Japan) with a load of 300 g and an indentation time for 10 s. The results were recorded as Vickers hardness number (VHN). The adopted hardness value of each specimen was the average of values obtained from 4 areas on the surface.

### 2.4. Shear Bond Strength 

The collection of extracted human teeth was approved by the Ethical Review Sub-Committee for Research Involving Human Research Subjects of Thammasat University, Thailand (ID: 008/2563; approval date: 11 August 2020). Forty extracted third molars with no visible caries were obtained from the Oral Health Department, Thammasat University Hospital, Pathum Thani, Thailand. The teeth were kept in 0.1% thymol solution at 25 °C for less than 3 months prior the test. 

The teeth were fixed in self-cured acrylic resin (n = 10). The coronal portion of the teeth was horizontally cut at 1–2 mm below the occlusal surface using a diamond disc under a cutting machine (Accutom 50, Struers, Cleveland, OH, USA) to expose the dentin surface. The cut surface was polished to standardize the smear layer using 500-grit silicon carbide paper for 60 s (Tegramin, Struers, Cleveland, OH, USA). The plastic tube (3 mm in diameter and 4 mm in height) was placed on the moist dentin surface to limit the bonding interface. The materials were prepared and placed into the tube. They were then light-cured for 40 s. The tip of the LED light-curing unit was positioned 1 mm above the tube. The specimens were left at room temperature for 24 h at 100% humidity to allow for complete setting. Then, the plastic was removed, and the specimens were placed in the shear bond strength testing jig. A metal blade was positioned at the interface of the specimen and dentin. The test was then performed under a mechanical testing frame (AGSX, SHIMADZU Corporation, Kyoto, Japan) using a 50 N load cell and a crosshead speed of 0.5 mm/min. Shear bond strength (SBS, Pa) was calculated using the following equation:

(1) $$\mathrm{SBS}=\frac{F}{A}$$

where F is the load at failure (N) and *A* is the area of bonding interface (mm^2^). The failure mode at the tooth–composite interface was analyzed under a stereomicroscope (Leica Zoom 2000, Leica Microsystems GmbH, Wetzlar, Germany). The representative specimens from each failure mode were randomly selected. They were sputter-coated with gold using a sputter coating machine (Q150R, Quorum Technologies, East Sussex, UK) with a current of 23 mA for 45 s. The bonded interface was then examined using a scanning electron microscope (SEM, JSM 7800F, JOEL, Tokyo, Japan) with an accelerated voltage of 5 kV. The mode of failure was classified as follows [26]:

(1) Adhesive failure between material and dentin.
(2) Cohesive failure mode within material.
(3) Mixed failure mode with both cohesive and adhesive failure.

### 2.5. Statistical Analysis

Values reported in the current study are mean ± SD. The data were analyzed using Prism 9.2 (GraphPad Software LLC., San Diego, CA, USA). The normality of data was checked using the Shapiro–Wilk test. For viscosity, gelation time, and surface microhardness, one-way ANOVA followed by post hoc Tukey multiple comparison was used to analyze the results. For SBS results, the Kruskal–Wallis test followed by Dunn’s multiple comparison was employed. The significance level was set at *p* = 0.05. Power analysis was performed using G*Power 3.1 software (University of Dusseldorf, Germany) [27]. The effect sizes (Cohen’s f) were calculated from rheological, microhardness, and SBS results in a pilot study. In order to obtain power greater than 0.99 in a one-way ANOVA (*α* = 0.05), G*Power suggested that sample sizes of 5, 5, and 10 for each group were required for the rheological, microhardness, and SBS tests, respectively.

## 3. Results

### 3.1. FTIR Studies

**Figure 4:**
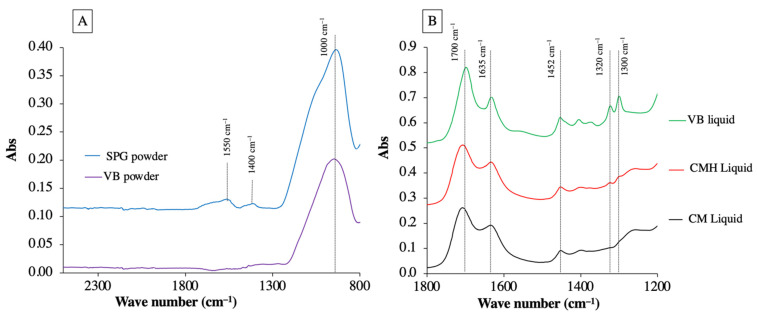
FTIR spectra of (**A**) powder and (**B**) liquid phases of RMGICs used in the current study.

Peaks attributable to the formation of polyacrylic salts (symmetric COO-stretch, 1470–1400 cm^−1^; asymmetric COO-stretch, 1600–1500 cm^−1^) were detected in SPG fillers, indicating the pre-reaction of glass and liquid (Figure 4). Additionally, the peaks representing the methacrylate group (1300 cm^−1^ and 1320 cm^−1^, C–O stretch) were detected with VB, CMH, and CM liquids. However, the absorbance of the C–O peak of CM and CMH was weaker than that of VB. FTIR peaks at ~1700–1720 cm^−1^ (C=O stretch, methacrylate group, and polyacids), 1635 cm^−1^ (C=C stretch of methacrylate group and O-H stretch of water), and 1452 cm^−1^ (C–H scissor, polyacids, and methacrylate group) were also detected [28,29].

### 3.2. Rheological Properties

**Figure 5:**
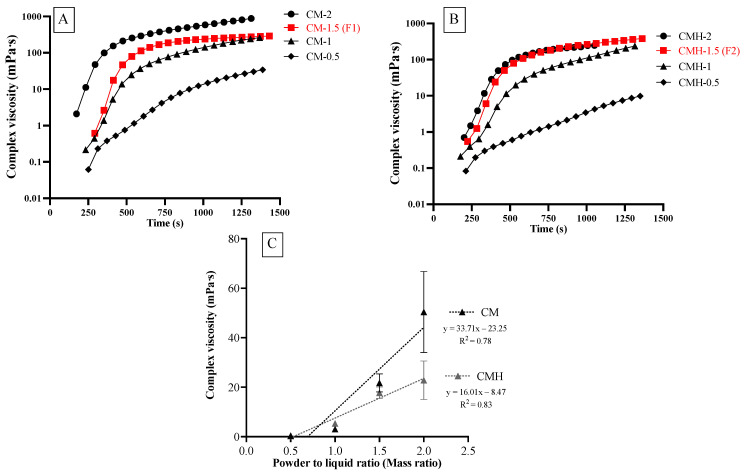
Viscosity of representative specimen obtained from the experimental RMGICs mixed with (**A**) CM and (**B**) CMH liquids using the powder-to-liquid ratios (PLRs) of 0.5:1, 1:1, 1.5:1, and 2:1. (**C**) mean viscosity of all groups immediately after mixing. Error bars are SD (n = 5).

After mixing, the viscosity of the experimental was increased with time (Figure 5A,B). Additionally, the viscosities of experimental materials increased linearly upon the increase in PLR (Figure 5C). The highest viscosity was detected with CM-PLR2 (50.4 m Pa·s) followed by CMH-PLR2 (22.9 m Pa·s). The viscosities of materials mixed with PLR of 0.5:1 (0.3–0.4 m Pa·s), 1:1 (3.1–5.3 m Pa·s), and 2:1 (22.8–50.4 m Pa·s) were difficult to handle during the test. Hence, the PLR of 1.5:1 (F1: CM-PLR1.5; F2: CMH-PLR1.5) was selected as the working PLR for the experimental RMGICs. 

**Figure 6:**
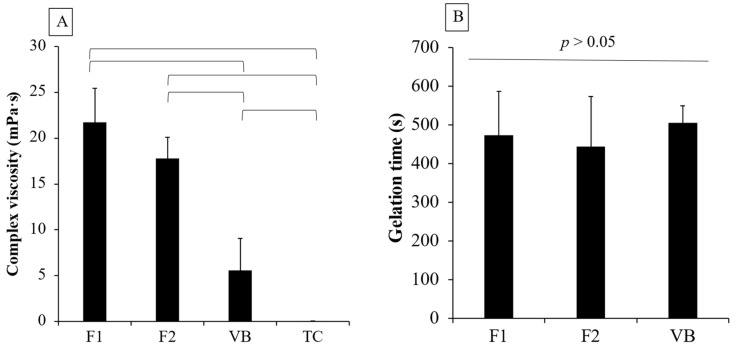
(**A**) viscosity of experimental RMGICs mixed with PLR of 1.5:1 using CM liquid (F1), CMH liquid (F2), and VB mixing, and TC after injecting from the syringe. (**B**) the gelation times of F1, F2, and VB, which are the times when the storage modulus (solid-like behavior) became equal and larger than the loss modulus (liquid-like behavior). The lines indicate *p* < 0.05. Error bars are SD (n = 5).

F1 (21.7 ± 3.7 m Pa·s) showed a comparable viscosity to that of F2 (17.8 ± 2.3 m Pa·s) (*p* = 0.1525) (Figure 6A). The viscosity of both F1 and F2 was significantly higher than that of VB (5.6 ± 3.5 m Pa·s) and TC (0.05 ± 0.01 m Pa·s) (*p* < 0.01). VB showed significantly higher viscosity than that of TC (*p* = 0.0286). Additionally, the gelation times of F1 (473.5 ± 112.7 s), F2 (443.3 ± 129.6 s), and VB (505.6 ± 43.4 s) were comparable (*p* > 0.05) (Figure 6B). It was not possible to measure the gelation time of TC, because the rheological properties of TC remained unchanged during the test.

### 3.3. Vickers Surface Microhardness 

**Figure 7:**
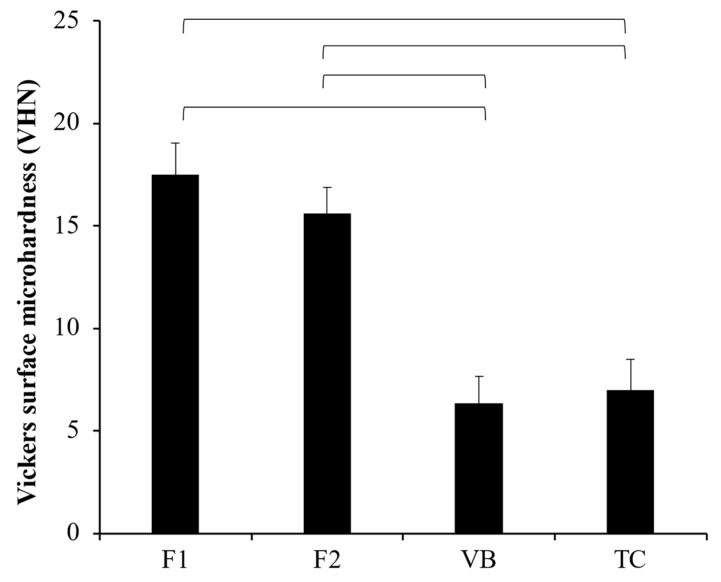
Vickers surface microhardness (VHN) of all materials after immersion in deionized water for 24 h. The lines indicate *p* < 0.05. Error bars are SD (n = 5).

The highest and lowest values of Vickers microhardness were obtained from F1 (17.5 ± 1.5 VHN) and VB (6.3 ± 1.3 VHN) (Figure 7). The hardness value of F1 was comparable to that of F2 (15.6 ± 1.3 VHN) (*p* = 0.1874). VB showed a similar surface microhardness to that of TC (7.0 ± 1.5 VHN) (*p* = 0.8776). Additionally, the surface microhardness of both F1 and F2 was significantly higher than that of VB and TC (*p* < 0.01). 

### 3.4. Shear Bond Strength (SBS)

**Figure 8:**
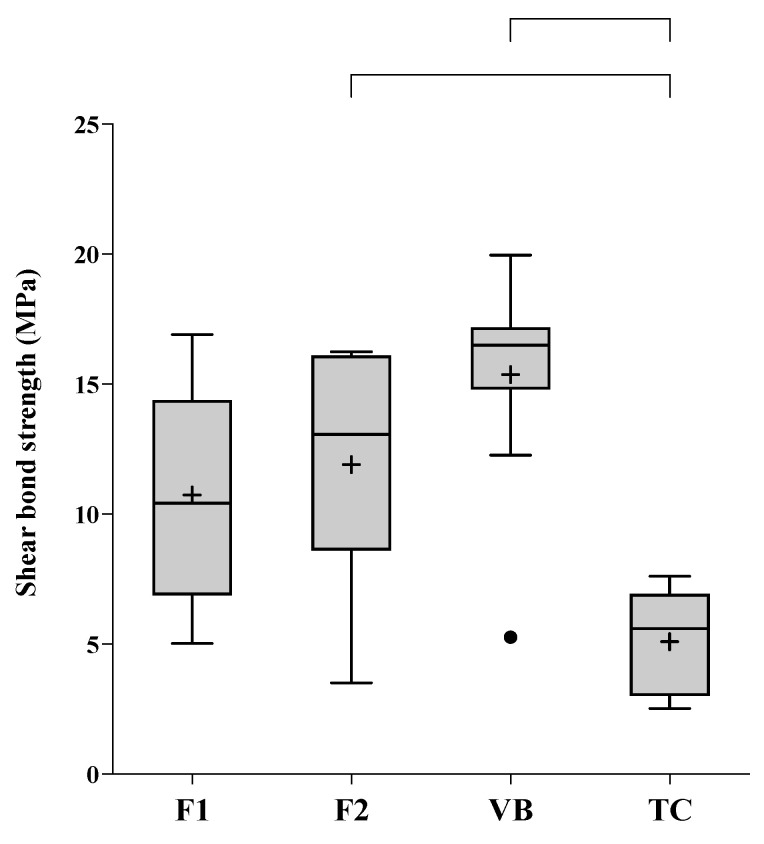
Shear bond strength to dentin of experimental RMGICs (F1 and F2) and commercial pulp protection materials. The boxes represent the first quartile (Q1) to the third quartile (Q3); the horizontal lines in the box represent the median; the whiskers represent the maximum and minimum values; and “+” represents the mean value (n = 10). The circle is an outlier. The lines indicate *p* < 0.05 (n = 10).

**Figure 9:**
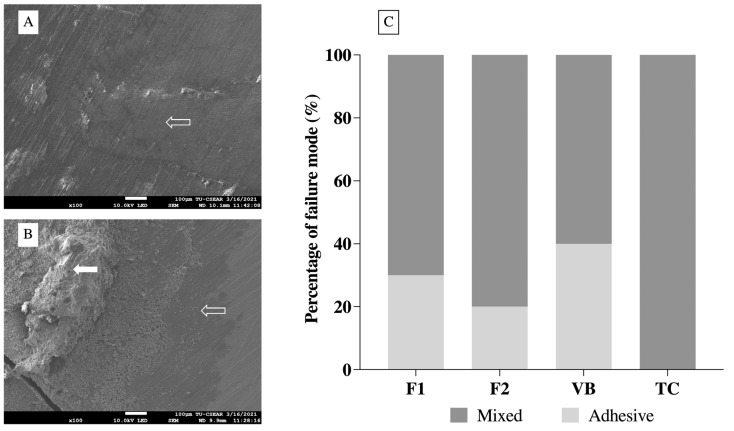
SEM images of debonded surface along the dentin side. (**A**) example of adhesive failure detected with the representative specimen randomly selected from the TC group. The specimen demonstrated the adhesively failed region (dentin exposure) between the material and dentin (unfilled arrow). (**B**) example of adhesive failure detected with the representative specimen from F1. The cohesively failed region (filled arrow) and the adhesively failed region (unfilled arrow) were observed. (**C**) percentage of failure modes observed from all groups (n = 10).

The highest and lowest SBSs (median, min–max) were obtained from VB (16.5, 5.3–20.0 MPa) and TC (5.6, 2.5–7.6 MPa) (Figure 8). Additionally, the SBS of VB was comparable to that of F1 (10.42, 5.0–16.9 MPa) (*p* = 0.2225) and F2 (10.42, 3.5–16.2 MPa) (*p* = 0.5766). The SBS of F1 was not significantly different from that of TC (*p* = 0.1118). The SBS of TC was, however, significantly lower than that of F2 (*p* = 0.0333) and VB (*p* < 0.01). The most common modes of failure detected with F1, F2, and VB were mixed failure followed by adhesive failure (Figure 9). The mode of failure observed with TC was all mixed failure. Cohesive failure was not detected in all groups. The distribution of failure modes between groups was not significantly different (*p* = 0.1705). 

## 4. Discussion

Resin-modified glass ionomer cements (RMGICs) are the most commonly used pulp protection materials for the restoration of deep carious cavity. The major concern regarding the current material is the potential toxic effects of unreacted HEMA on human cells. The current study prepared RMGICs for pulp protection using polyacids functionalized with methacrylate groups to reduce the need for HEMA. The liquid was mixed with spherical pre-reacted glass fillers. The effects of using two different liquid phases, CM liquid (0 wt. % HEMA) or CMH liquid (5 wt. % HEMA), on the rheological properties, shear bond strength to dentin, and Vickers microhardness of the experimental RMGICs were assessed. 

The results demonstrated that the use of different liquid phases showed negligible effects on the rheological properties, shear bond strength to dentin, and Vickers microhardness of the experimental materials. Hence, there was insufficient evidence to reject the first null hypothesis. However, the second hypothesis was rejected, as the experimental RMGICs showed significantly different viscosity and surface microhardness compared to that of the commercial RMGIC.

### 4.1. FTIR Studies 

The FTIR spectra of the SPG fillers revealed the peaks that represent acid-base reaction, confirming the presence of pre-reaction components in the SPG fillers. Additionally, the peaks attributable to the methacrylate groups (1320 and 1300 cm^−1^) were detected with CM and CMH. However, the intensity of the absorbance was much lower than that obtained from VB, as was expected. This could be due to the lower methacrylate groups and low level of HEMA (5 wt. %) compared to that of VB (20–30 wt. %).

### 4.2. Rheological Properties 

Pulp protection materials should exhibit low viscosity to promote good flow and adaptation of materials with an irregular cavity floor. This may help reduce the risk of gaps at the tooth–material interface, which could lead to post-operative sensitivity or leakage of the restoration [30]. Additionally, excessive viscosity of the material may detrimentally affect handling properties, which could complicate the filling procedures for clinicians. However, it should be mentioned that the viscosity and gelation time required by the standards are not yet specified. 

Initially, we characterized the viscosity of the experimental RMGICs using different PLRs ranging from 0.5:1 to 2:1 to obtain the optimum mixing ratio. The increase in viscosity of RMGICs after mixing in the current study could be due to the materials undergoing free radical polymerization and acid-base neutralization [11]. The lowest PLR gave the lowest viscosity, whilst the highest PLR exhibited the highest viscosity, as was expected. The use of high PLR generally led to an increase in mechanical strength, but it also shortened the working and setting time for glass ionomer materials [22,31]. Additionally, the excessive high or low viscosity of materials may compromise their handling characteristics. The PLR of 1.5:1 was selected due to its acceptable handling properties. The mixing ratio was also similar to that of Vitrebond (PLR of 1.4:1). The gelation time of RMGICs may represent the time point when the flowability of material was reduced due to the material’s transition from liquid-like to solid-like behavior [32]. This may affect the material’s handling or loading into the cavity. The comparable gelation time of the experimental materials with VB may facilitate the use of new materials for clinicians. We speculate that RMGICs with low viscosity may exhibit a longer gelation time than that of materials with higher viscosity. However, the gelation time of VB was comparable to that of the experimental RMGICs. A possible explanation could be that VB may exhibit a faster acid-base reaction. Another reason could be that HEMA monomers in VB were polymerized upon exposure to natural light. This may subsequently lead to a rapid increase in the storage modulus of VB.

The use of 2-hydroxyethyl methacrylate (HEMA, viscosity ~6.79 m Pa·s) could help to reduce the viscosity of RMGICs. The results of the current study indicate that the addition of 5 wt. % HEMA exhibited minimal effects on the rheological properties of the materials. The viscosities of F1 and F2 were higher than that of VB. The reason could be that the liquid of VB consisted of a higher level of HEMA (20–30 wt. %) than that of the experimental RMGICs. Additionally, the smaller particle size of SPG (particle size of 10–20 μm) compared with that of VB powder (particle size of 8–40 μm [33]) may cause the faster setting of experimental RMGICs [34]. The assessment of degree of monomer conversion upon light-curing should be included in future studies.

### 4.3. Surface Microhardness

For pup protection materials, high mechanical properties, such as surface microhardness, are essential to ensure that they can withstand the cavity drilling or acid etching during the placement of definitive restorative materials [35,36]. The mechanical properties of glass ionomer cements are governed by various factors, such as the different powder-to-liquid ratio [22], particle size and shape [34], and composition of powder and liquid phases [21,37].

The lowest surface microhardness value was detected with TC, which could be due to the high hydrophilicity of Ca-Si cement [38] enhancing water sorption into the material. The experimental RMGICs showed higher surface microhardness than that of Vitrebond. This could be due to the following reasons: firstly, the high level of HEMA in VB may encourage water sorption plasticizing the polymer matrix [39], thus reducing the hardness of materials; secondly, the use of pre-reacted glass with small particle size may promote polymer cross-linking which could increase the strength of the materials [23,40]. Additionally, high molecular weight polyacids of CM liquid may help to increase rigidity of the polymer network in the experimental RMGICs [41]. Additionally, the slightly higher PLR used in the experimental RMGICs (1.5:1 versus 1.4:1) compared with VB may enable a higher level of remaining filler after acid-base reaction. A high level of a reinforcing phase is associated with an increase in brittleness of materials, which may subsequently enhance their surface microhardness [42]. The result also indicated that the addition of HEMA at 5 wt. % showed no detrimental effect on surface hardness of the material. This could be due to the fact that the low level of HEMA showed no detrimental effect on mechanical properties of the materials.

### 4.4. Shear Bond Strength

Pulp protection materials should exhibit the ability to adhere to dentin and seal dentinal tubules [35]. Strong adhesion to dentin is crucial to prevent leakage at the tooth-restoration interface that may ultimately lead to pulpal infection and failure of treatment [43]. The effective adhesion of materials requires good wetting of the substrate and mechanical retention or chemical bonding between the materials and tooth structure [44].

Currently, the minimum SBS required by RMGICs used for pulp protection material is not specified by ISO standards. However, the obtained SBS of the experimental RMGICs in the current study (10–17 MPa) was in the range of that detected with the commercial materials (6–20 MPa) [45,46,47,48,49]. Although the SBS obtained from the experimental RMGICs was not significantly different from that of VB, the highest observed mean SBS was detected with VB. This could be due to the fact that the high level of HEMA in VB promoted material adaptation and penetration into moist dentin. This could potentially enhance the mechanical interlocking in the hybrid layer and subsequently increase the bond strength to dentin [45]. It should be mentioned that the reproducibility of SBS testing was generally low. The high variability of the data could be influenced by different dentin substrates, levels of mineralization, bonding areas, and the depth of dentin in each tooth [50,51]. This was reflected by the fact that the larger sample size for SBS testing was needed to ensure that the high statistical power (>0.99) can be obtained.

The current study demonstrated that mixed failure was the most common mode of failure observed with RMGICs, which is in an agreement with the results of published studies [9,45,52]. This may be due to the presence of chemical/mechanical interactions between the materials and dentin [12,53]. Low SBS detected with TC could be due to the reduction of light transmission by opaque calcium silicate cement in TC. This may subsequently decrease the polymerization of TC at the interface [54]. Additionally, the use of high molecular weight methacrylate monomer (polyethylene glycol dimethacrylate, MW = 198.22 g/mol) in TC may reduce radical chains mobility [9], thus reducing polymer crosslinks in the material. The SBS of TC in the current study (2.5–7.6 MPa) was higher than that reported in the published study (1.14 ± 0.63 [9], 0.09 ± 0.20 [55]). A possible explanation could be that the current study employed a longer curing time (40 s) than that used in the published studies (20 s). The extended curing time could potentially enhance the free-radical polymerization and strength of the material [56,57]. The limitation of the current preliminary study is that the SBS measurement was performed only at an early time. Future studies with a longer aging time may be required to assess the bond durability of the materials.

## 5. Conclusions

The experimental RMGICs prepared by mixing polyacid acids functionalized with methacrylate groups and pre-reacted spherical glass fillers showed physical and mechanical properties comparable to those of the commercial RMGIC. The effect of incorporating HEMA (5 wt. %) into the acidic liquid showed no significant effect on rheological properties, surface microhardness, or shear bond strength of the experimental RMGICs. These experimental materials could be considered as alternative pulp protection materials.

## Figures and Tables

**Table 1:** Composition of liquid phases used in the current study.

Liquid Formulations	Composition
CM	CM polymer (55 wt. %), water (45 wt. %), tartaric acid (2 pph ^1^), camphorquinone (0.7 pph), *N*,*N*′-dimethylaminoethyl methacrylate (1.4 pph)
CMH	CM polymer (50 wt. %), water (45 wt. %), 2-hydroxyethyl methacrylate (5 wt. %), tartaric acid (2 pph), camphorquinone (0.7 pph), *N*,*N*′-dimethylaminoethyl methacrylate (1.4 pph)

^1^ parts per hundred.

**Table 2:** Composition of commercial materials used in the current study.

Materials	Composition	Instruction	Suppliers	Lot No.
Vitrebond (VB)	Powder: glass powder (>95 wt. %), diphenyliodonium chloride (<2 wt. %) Liquid: copolymer of acrylic and itaconic acids (35–45 wt. %), 2-hydroxyethyl methacrylate (20–30 wt. %), water (30–40 wt. %) Powder-to-liquid ratio: 1.4:1 (mass ratio)	- Dispense 1 level of powder scoop and 1 drop of liquid - Mix within 10–15 s - Light-cure for 30 s	3M ESPE, St. Paul, MN, USA	BN981834
TheraCal LC (TC)	Calcium-silicate cement (30–50 wt. %), polyethylene glycol dimethacrylate (10–30 wt. %), barium zirconate powder (1–10 wt. %)	- Inject material from the syringe - Light-cure for 20 s	Bisco Inc., Schaumburg, IL, USA	1900006662

## Data Availability

The datasets generated during and/or analyzed during the current study are available from the corresponding author on reasonable request.
